# Tenosynovial Giant Cell Tumor Observational Platform Project (TOPP) Registry: A 2-Year Analysis of Patient-Reported Outcomes and Treatment Strategies

**DOI:** 10.1093/oncolo/oyad011

**Published:** 2023-03-03

**Authors:** Emanuela Palmerini, John H Healey, Nicholas M Bernthal, Sebastian Bauer, Hendrik Schreuder, Andreas Leithner, Javier Martin-Broto, Francois Gouin, Julio Lopez-Bastida, Hans Gelderblom, Eric L Staals, Florence Mercier, Petra Laeis, Xin Ye, Michiel van de Sande

**Affiliations:** IRCCS Istituto Orthopedico Rizzoli, Bologna, Italy; Memorial Sloan Kettering Cancer Center, New York, NY, USA; David Geffen School of Medicine at UCLA, Santa Monica, CA, USA; West German Cancer Center, University of Duisburg-Essen, Essen, Germany; Radboud University, Nijmegen, The Netherlands; Department of Orthopaedics and Trauma, Medical University of Graz, Graz, Austria; Fundacíon Jiménez Díaz University Hospital, ATBSARC lab in General Hospital of Villalba, IIS-FJD, Madrid, Spain; Centre Léon Bérard, Lyon, France; University Castilla-La Mancha, Talavera de la Reina, Spain; Leiden University Medical Center, Leiden, The Netherlands; IRCCS Istituto Orthopedico Rizzoli, Bologna, Italy; Daiichi Sankyo Europe GmbH, Munich, Germany; Daiichi Sankyo Europe GmbH, Munich, Germany; Daiichi Sankyo, Inc., Basking Ridge, NJ, USA; Leiden University Medical Center, Leiden, The Netherlands

**Keywords:** diffuse-TGCT, pexidartinib, patient-reported outcome (PRO), prospective, quality of life (QoL), tenosynovial giant cell tumor observational platform project (TOPP)

## Abstract

**Background:**

The Tenosynovial giant cell tumor Observational Platform Project (TOPP) registry is an international prospective study that ­previously described the impact of diffuse-type tenosynovial giant cell tumour (D-TGCT) on patient-reported outcomes (PROs) from a baseline snapshot. This analysis describes the impact of D-TGCT at 2-year follow-up based on treatment strategies.

**Material and Methods:**

TOPP was conducted at 12 sites (EU: 10; US: 2). Captured PRO measurements assessed at baseline, 1-year, and 2-year follow-ups were Brief Pain Inventory (BPI), Pain Interference, BPI Pain Severity, Worst Pain, EQ-5D-5L, Worst Stiffness, and ­Patient-Reported Outcomes Measurement Information System. Treatment interventions were no current/planned treatment (Off-Treatment) and systemic treatment/surgery (On-Treatment).

**Results:**

A total of 176 patients (mean age: 43.5 years) were included in the full analysis set. For patients without active treatment strategy ­(Off-Treatment) at baseline (*n* = 79), BPI Pain Interference (1.00 vs. 2.86) and BPI Pain Severity scores (1.50 vs. 3.00) were numerically favorable in patients remaining Off-Treatment compared with those who switched to an active treatment strategy at year 1. From 1-year to 2-year ­follow-ups, patients who remained Off-Treatment had better BPI Pain Interference (0.57 vs. 2.57) and Worst Pain (2.0 vs. 4.5) scores compared with patients who switched to an alternative treatment strategy. In addition, EQ-5D VAS scores (80.0 vs. 65.0) were higher in patients who remained ­Off-Treatment between 1-year and 2-year follow-ups compared with patients who changed treatment strategy. For patients receiving systemic treatment at baseline, numerically favorable scores were seen in patients remaining on systemic therapy at 1-year follow-up: BPI Pain Interference (2.79 vs. 5.93), BPI Pain Severity (3.63 vs. 6.38), Worst Pain (4.5 vs. 7.5), and Worst Stiffness (4.0 vs. 7.5). From 1-year to 2-year follow-up, EQ-5D VAS scores (77.5 vs. 65.0) were higher in patients who changed from systemic treatment to a different treatment strategy.

**Conclusion:**

These findings highlight the impact D-TGCT has on patient quality of life, and how treatment strategies may be influenced by these outcome measures. (ClinicalTrials.gov number: NCT02948088)

## Teaser

The TOPP registry is an international prospective study that previously described the impact of diffuse-type tenosynovial giant cell tumor on patient-reported outcomes from a baseline snapshot. This article reports a 2-year follow-up based on treatment strategies and could represent a benchmark for future clinical trials.

**Implications for Practice**

These findings extend beyond what has been previously published, as this analysis is the first to describe the impact of D-TGCT on PRO as a prospective 2-year follow-up based on treatment strategies and could represent a benchmark for future clinical trials. As optimal treatment strategies remain to be elucidated, the treatment of D-TGCT patients is multidisciplinary and multimodal. The practical use of PRO assessments for treatment evaluation in this rare disease needs to be further evaluated in future studies.

## Introduction

Tenosynovial giant cell tumor (TGCT) is a rare, locally aggressive mesenchymal neoplasm arising from the synovium of joints, bursae, and tendon sheaths, associated with colony-stimulating factor 1 (CSF1) overexpression.^1^ It affects small and large joints (mainly the knee), and symptoms include pain, stiffness, swelling, and limited range of motion.^2,3^ Two subtypes of TGCT are defined based on clinical and radiological characteristics: localized- and diffuse-type TGCT (L-TGCT and D-TGCT).^4,5^ Both subtypes share a common pathophysiology, and they represent a wide spectrum of clinical entities, making TGCT behavior complex and hard to predict.^6^ D-TGCT constitutes 10%–20% of all TGCT cases, usually occurring in large joints (eg, knee, ankle, and hip), is an aggressive multi-lobulated lesion located intra- and/or extra-articular, and has a detrimental effect on the quality of life (QoL).^7-11^

A proper diagnosis of TGCT can take several years due to the non-specific symptoms and the rarity of this disease, resulting in severely delayed optimal treatment and care and a higher risk of insufficient treatment or undertreatment for these patients.^9,12^ The current standard of care for patients with TGCT is surgical resection of the tumor as completely as possible to reduce symptoms and prevent joint destruction, improve function, and minimize the risk of recurrence.^13^ While surgery cures the vast majority of L-TGCT cases, D-TGCT shows a high tendency toward local recurrence, occurring in over half of the resected cases. Therefore, the value of surgery in the D-TGCT subtype is variable.^14-16^

The CSF1 receptor (CSF1R) has been the target of promising therapies.^17-19^ Pexidartinib has been approved by the US Food and Drug Administration at a dosage of 400 mg twice daily, and the National Comprehensive Cancer Network added it as a category 1 recommendation for the treatment of adult patients with severe symptomatic TGCT associated with severe morbidity or functional limitations and not amenable to improvement with surgery.^20,21^ Pexidartinib is only available through a restricted program under a Risk Evaluation and Mitigation Strategy (REMS), because of the risk of hepatotoxicity, and was not approved by the European Medicines Agency. Other non-registered systematic therapies have been used either off-label or in clinical trials (ie, imatinib ­[off-­label], nilotinib [trial and off-label], emactuzumab [trial], and cabiralizumab [trial]).^3,14,15,22,23^

To date, most epidemiologic understanding of D-TGCT comes from small, retrospective studies that traditionally focused on oncological outcomes.^1,24^ Questions to elaborate the true morbidity and actual impact on QoL of both the disease and its various treatment options remain to be elucidated.

Presently, there are few data available detailing the management of patients with TGCT, the disease effect of TGCT for patients (including pain, joint stiffness, swelling, reduced mobility, and QoL) or the economic impact of TGCT. ­Patient-Reported Outcomes Measurement Information System-Physical Function (PROMIS-PF) was developed in 2005 by the National Cancer Institute (NCI) to measure the impact of the disease on patients’ ability to perform daily activity through self-administered items and validated for patients with TGCT in 2019.^8^

Previously, the prospective international TGCT Observational Platform Project (TOPP) registry described the impact of TGCT on patient-reported outcomes (PROs) from a baseline snapshot.^25^ In addition, a more recent analysis of this registry provided a picture of the treatment journey of D-TGCT patients as a 2-year observational follow-up.^26^ This analysis is the first to describe the impact of the disease on PROs at a 2-year follow-up based on treatment strategies.

## Materials and Methods

### Study Design and Participants

This global multicenter, prospective sponsored study included all consecutive patients from 12 tertiary sarcoma centers in 7 EU countries and 2 US sites. Key eligibility criteria and study designs for the TOPP registry have been described elsewhere.^25^

Briefly, patients aged ≥18 years had primary or recurrent D-TGCT. Recurrent disease was defined as tumor recurrence after complete resection or progression of the residual tumor.^25^ While watchful waiting was a common treatment strategy, systemic treatment was preferred for patients with primary disease, whereas patients with recurrent disease often were treated with surgery and other treatment modalities.^26^ TGCT was histologically confirmed and assessed as diffuse based on magnetic resonance imaging (MRI) or clinical presentation, if MRI was missing. The observation period time per patient was 24 months (± 2 months).

**Figure 1.**
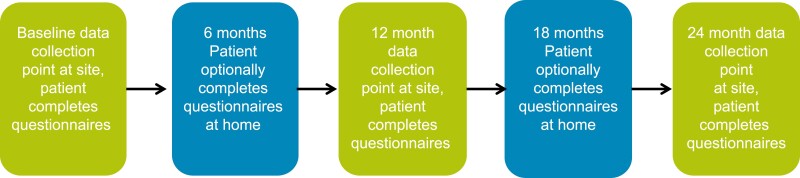
Study design. *Additional data collection points may have occurred at any time the patient visited the site, even if it was outside this schedule.

PRO measurements were captured at baseline, 6 months, 1 year, 18 months, and 2 years. For this analysis, PROs taken at baseline (at time of enrollment in the registry), and at the 1-year and 2-year follow-up collection points were used (Fig. 1).

Patients were followed prospectively, and data were collected based on the type of treatment strategy. Patients who were actively surveilled during the 2-year observation period were not actually being treated and did not have a planned treatment were categorized as Off-Treatment (*n* = 79). Patients undergoing active intervention (eg, surgery, systemic therapy) were classified as On-Treatment (*n* = 97). Of these 97 patients, 84 (either systemic therapy only [*n* = 45] or surgery only [*n* = 39]) were included in this analysis. The other 13 patients (radiotherapy [*n* = 5], future surgery [*n* = 4], surgery + systemic [*n* = 2], surgery + ^90^Yttrium [*n* = 1], and systemic + future surgery [*n* = 1]) were not included as the patient populations were regarded too small to analyze.

Patients were considered “On-Treatment” if undergoing surgery (at baseline, or within the 2-year observation period), and if undergoing systemic treatment (at baseline or within the 2-year period). Furthermore, patients undergoing surgery at baseline were also considered “On-Treatment” within the 2-year observation period as surgery does not entail a procedure at each time point (baseline, 1 year, and 2 years).

PRO endpoints were calculated as medians of all available scores collected at each time point for each patient. Only patients who filled out the PRO questionnaires at a specific time point (baseline, 1 year, or 2 years) were included in the analysis. The following were assessed:

### Brief Pain Inventory (BPI)

Developed by the Pain Research Group Anderson Cancer Center, University of Texas, the BPI rapidly assesses the severity of pain and its impact on daily functioning. It measures the impact of pain on daily function, location of pain, pain medications, and amount of pain relief in the past 24 h. The BPI gives 2 main scores: a pain severity score and a pain interference score. The BPI Pain Severity scale (rated from 0 = no pain to 10 = pain as bad as you can imagine) is calculated from 4 items about pain intensity; BPI Pain Interference scale (rated from 0 = does not interfere, to 10 = completely interferes) corresponds to the item on pain interference with 7 sub-items. Also, the BPI Worst Pain scale (rated from 0 = no pain to 10 = pain as bad as you can imagine) rates the patient’s pain by circling a number (0-10) that best describes their pain at its worst over the last 24 h.

### EQ-5D-5L

The first part consists of 5 domains (mobility, self-care, usual activities, pain or discomfort, and anxiety or depression). Each domain has 5 levels ranging from “no problems” through “profound difficulties” with a sum utility score ranging from 0 to 1. In the second part, patients have to rate their current health on a 20-cm vertical visual analog scale (VAS; 0-100 with 0 = worst health that you can imagine and 100 = best health that you can imagine).

### Worst Stiffness

A single-item (from 0 to 10 with 0 = no stiffness and 10 = stiffness as bad as you can imagine) numerical rating scale.

### Patient-Reported Outcomes Measurement Information System (PROMIS)

PROMIS Physical Function (PROMIS-PF) rating scale (patients with lower extremity tumors evaluated 13 items assessing lower limb function; patients with upper extremity tumors evaluated 11 items assessing upper limb function; scale 0–100). More specifically, the results from both sets (upper extremity and lower extremity) were combined and analyzed together. Each item (ie, upper extremity: “Are you able to change a light bulb overhead?” and lower extremity: “Are you able to go for a walk of at least 15 min?” had 5 response options ranging in value from 1 [task cannot be performed] to 5 [task can be performed without difficulties]). Scores were expressed as *T*-scores, which are standardized to a mean of 50 and a standard deviation (SD) of 10, where a higher score signified better physical function. Calculation of PROMIS scores was performed according to the scoring procedure by the Health Measures Scoring Service.^27^

Patients On-Treatment at baseline, followed by the same type of treatment or wait-and-see at years 1 and/or 2 were documented as “remaining on the same treatment.” Treatment interventions at years 1 and/or 2 were classified as ­Off-Treatment, systemic treatment only (pexidartinib, ­imatinib, nilotinib, investigator study medicine), surgery only, radiotherapy or ^90^Yttrium, systemic treatment + other (radiotherapy or ^90^Yttrium, or surgery), or surgery + other ­treatment (radiotherapy or ^90^Yttrium, or systemic treatment).

### Statistical Analysis

Binary, categorical, and ordinal parameters have been summarized by means of absolute and percentage numbers within the various categories (including “missing data” as a valid category at baseline). Numerical data were summarized using standard statistics (ie, number of available data, number of missing data, median, SD, and minimum, median, maximum, and lower and upper quartiles).

## Results

### Subjects

A total of 183 patients enrolled in the all-document patient set (APS) fulfilled the inclusion criteria and were available for baseline analysis, resulting in identical APS and baseline analysis set (BAS) datasets. Of the 183 patients from BAS, 4 patients withdrew their informed consent, and no post-baseline documented information was available for 3 patients, hence the full analysis set (FAS) is based on the data of the remaining 176 patients and is the population used in this analysis (Supplementary Fig. S1).

Most patients (120/176 [68.2%]) had a knee tumor, and 108/176 (61.4%) of the patients were female. The mean (±SD) age at enrollment was 43.5 ± 14.29 years. Baseline demographics are summarized in Supplementary Table S1. At baseline, 97/176 patients were On-Treatment (actively treated), whereas 79/176 were Off-Treatment (wait-and-see). Specific treatment plans at baseline are summarized in Supplementary Fig. S2.

At baseline, among 176 patients in the FAS, 47 patients (26.7%) either received (*n* = 42) or planned to receive surgery (*n* = 5), 48 patients (27.4%) received or planned to receive systemic therapy, and 6 patients (3.4%) received (*n* = 5) or planned to receive radiotherapy (*n* = 1; including ^90^Yttrium therapy; Table 1).

**Table 1.** Type of TGCT treatment plan during the observation period.

Type of treatment, *n* (%)	Baseline (*N* = 176)	Within 1st year (*N* = 173)	Within 2nd year (*N* = 173)
Radiotherapy	5 (2.8)	1 (0.6)	0
^90^Yttrium therapy	1 (0.6)	1 (0.6)	0
Systemic	48 (27.3)	16 (9.2)	8 (4.6)
Surgery	42 (23.9)	40 (23.1)	25 (14.5)
Future surgery required	5 (2.8)	2 (1.2)	0
**Status of TGCT treatment**	**Baseline (*N* = 176)**	**Within 1st year (*N* = 173)**	**Within 2nd year (*N* = 173)**
Current	44 (25.0)	50 (28.9)	23 (13.3)
Planned	50 (28.4)	9 (5.2)	9 (5.2)
No treatment (Off-Treatment/follow-up)	79 (44.9)	77 (44.5)	105 (60.7)
**Type of current treatment during observation period**	**Baseline (*n* = 44)**	**Within 1st year (*n* = 50)**	**Within 2nd year (*n* = 23)**
Radiotherapy	0	0	0
^ 90^Yttrium therapy	0	1 (2.0)	0
Systemic	37 (84.1)	12 (24.0)	8 (34.8)
Surgery	7 (15.9)	37 (74.0)	17 (73.9)
**Type of planned treatment during observation period**	**Baseline (*n* = 50)**	**Within 1st year (*n* = 9)**	**Within 2nd year (*n* = 9)**
Radiotherapy	5 (10.0)	1 (11.1)	0
^ 90^Yttrium therapy	1 (2.0)	0	0
Systemic	11 (22.0)	4 (44.4)	0
Surgery	36 (72.0)	4 (44.4)	9 (100)

Percentages are based on the total number of patients who documented ≥1 current treatment (excluding patients with missing and unknown data).

Percentage calculation can sum to >100% because patients can have >1 treatment planned.

Abbreviation: TGCT, tenosynovial giant cell tumor.

A total of 44 patients (25%) were being actively treated at baseline (current treatment), of which 37 (84.1%) received systemic therapy (ie, pexidartinib, imatinib, nilotinib, or investigator study medicine) while the other 7 patients (15.9%) were treated with surgery (Table 1). Furthermore, treatment was planned for 50 (28.4%) patients, most were surgery (72%, *n* = 36), followed by systemic therapy (22%, *n* = 11), and radiotherapy (12%, *n* = 6; Table 1).

### QoL Assessments from Baseline Through 2 Years

#### No Treatment at Baseline (Off-Treatment)

**Figure 2.**
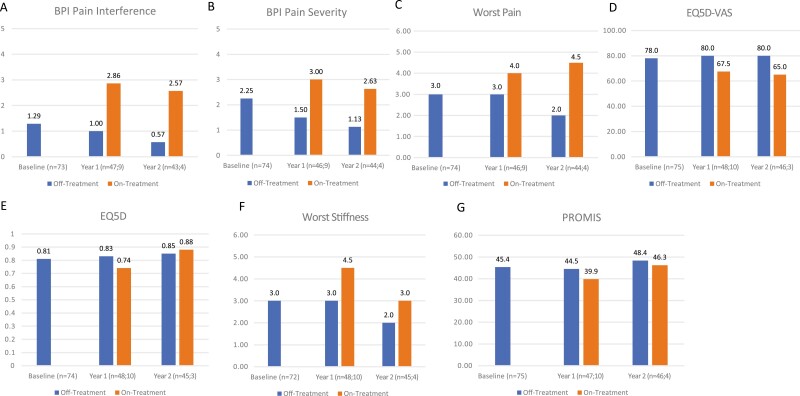
QoL for patients undergoing surgery at baseline, and changes at years 1 and 2 of observation assessed by the following PROs: (**A**) BPI Pain Interference; (**B**) BPI Pain Severity; (**C**) Worst Pain; (**D**) EQ-5D VAS; (**E**) EQ-5D; (**F**) Worst Stiffness; and (**G**) PROMIS scores. Calculated as median scores. BPI = brief pain inventory; EQ-5D VAS = EuroQol-5 Dimension visual analog scale; PRO = patient-reported outcome; PROMIS = Patient-Reported Outcomes Measurement Information System; QoL, quality of life.

This section compares patients who were Off-Treatment at baseline and remained Off-Treatment throughout the 2-year observation period versus patients who changed from Off-Treatment to On-Treatment (systemic treatment or surgery) at years 1 and/or 2. For patients who were Off-Treatment at baseline, numerically favorable scores (median values) were observed in patients who remained Off-Treatment compared with patients who switched to an active treatment strategy at year 1: median BPI Pain Interference (*n* = 47, 1.00 vs. *n* = 9, 2.86; Table 2; Fig. 2A), BPI Pain Severity (*n* = 46, 1.50 vs. *n* = 9, 3.00; Table 2; Fig. 2B), and Worst Stiffness (*n* = 48, 3.0 vs. *n* = 10, 4.5; Table 2; Fig. 2F). EQ-5D index score (*n* = 48, 0.83 vs. *n* = 10, 0.74; Table 2; Fig. 2D) and EQ-5D VAS (*n* = 48, 80.0 vs. *n* = 10, 67.5; Table 2; Fig. 2E). At year 2, median BPI Pain Interference (*n* = 43, 0.57 vs. *n* = 4, 2.57; Table 2; Fig. 2A), BPI Pain Severity (*n* = 44, 1.13 vs. *n* = 4, 2.63; Table 2; Fig. 2B) Worst Stiffness (*n* = 45, 2.0 vs. *n* = 4, 3.0; Table 2; Fig. 2F) Worst Pain (*n* = 44, 2.0 vs. *n* = 4, 4.5; Table 2; Fig. 2C), EQ-5D VAS (*n* = 46, 80.0 vs. *n* = 3, 65; Table 2; Fig. 2D) scores were numerically better in patients who remained Off-Treatment as compared with those who changed treatment. PROMIS scores were similar regardless of whether patients changed or remained on the same treatment strategy throughout the 2 years (Table 2; Fig. 2G).

**Table 2.** PROs for patients completing questionnaires at each time point: Off-Treatment, systemic treatment, and surgery.

Off-Treatment, baseline (*n* = 79)							
	BPI Pain Interference	BPI Pain Severity	Worst Pain	EQ-5D VAS	EQ-5D index	Worst Stiffness	PROMIS
Completed PROs	*n* = 73	*n* = 74	*n* = 74	*n* = 75	*n* = 74	*n* = 72	*n* = 75
Median	1.29	2.25	3.0	78.0	0.81	3.0	45.4
95% CI	0.57, 2.29	1.75, 2.75	2.0, 4.0	70.0, 80.0	0.77, 0.85	2.0, 4.0	42.4, 47.4
Q1, Q3	0.14, 4.00	0.75, 4.00	1.0, 6.0	60.0, 90.0	0.71, 0.91	1.5, 5.0	37.1, 49.9

1 year, remained Off-Treatment (*n* = 60)							
	BPI Pain Interference	BPI Pain Severity	Worst Pain	EQ-5D VAS	EQ-5D index	Worst Stiffness	PROMIS
Completed PROs	*n* = 47	*n* = 46	*n* = 46	*n* = 48	*n* = 48	*n* = 48	*n* = 47
Median	1.00	1.50	3.0	80.0	0.83	3.0	44.5
95% CI	0.14, 2.57	1.00, 2.75	2.0, 4.0	75.0, 85.0	0.80, 0.87	2.0, 4.0	40.7, 50.1
Q1, Q3	0, 3.43	1.00, 3.25	1.0, 5.0	70.0, 90.0	0.75, 0.91	1.0, 5.0	39.2, 53.0

Changed to On-Treatment (*n* = 11)							
	BPI Pain Interference	BPI Pain Severity	Worst Pain	EQ-5D VAS	EQ-5D index	Worst Stiffness	PROMIS
Completed PROs	*n* = 9	*n* = 9	*n* = 9	*n* = 10	*n* = 10	*n* = 10	*n* = 10
Median	2.86	3.00	4.0	67.5	0.74	4.5	39.9
95% CI	2.14, 7.43	2.25, 4.00	3.0, 7.0	65.0, 90.0	0.53, 0.91	1.0, 7.0	33.6, 56.0
Q1, Q3	2.71, 5.57	2.50, 3.25	3.0, 5.0	65.0, 80.0	0.66, 0.81	2.0, 7.0	33.8, 44.8
Unknown treatment (*n* = 8)							

2 years, remained Off-Treatment (*n* = 54)^a^							
	BPI Pain Interference	BPI Pain Severity	Worst Pain	EQ-5D VAS	EQ-5Dindex	Worst Stiffness	PROMIS
Completed PROs	*n* = 43	*n* = 44	*n* = 44	*n* = 46	*n* = 45	*n* = 45	*n* = 46
Median	0.57	1.13	2.0	80.0	0.85	2.0	48.4
95% CI	0.14, 1.43	0.75, 2.00	1.0, 3.0	75.0, 90.0	0.80, 0.91	2.0, 3.0	44.2, 50.2
Q1, Q3	0, 2.00	0.38, 2.88	0.5, 4.0	70.0, 95.0	0.77, 1	1.0, 4.0	40.8, 52.5

Changed to On-Treatment (*n* = 5)							
Completed PROs	*n* = 4	*n* = 4	*n* = 4	*n* = 3	*n* = 3	*n* = 4	*n* = 4
Median	2.57	2.63	4.5	65.0	0.88	3.0	46.3
95% CI	0, 6.43	0, 6.75	0, 7.0	0, 100	0.63, 1	0, 7.0	36.2, 51.6
Q1, Q3	0, 5.79	0.38, 5.63	1.0, 7.0	0, 100	0.63, 1	0, 6.5	40.1, 50.1

Systemic treatment only, baseline (*n* = 45)							
	BPI Pain Interference	BPI Pain Severity	Worst Pain	EQ-5D VAS	EQ-5Dindex	Worst Stiffness	PROMIS
Completed PROs	*n* = 42	*n* = 42	*n* = 42	*n* = 43	*n* = 42	*n* = 43	*n* = 43
Median	2.86	3.88	5.5	70.0	0.70	6.0	39.8
95% CI	1.71, 4.86	3.00, 5.00	4.0, 7.0	65.0, 75.0	0.61, 0.78	4.0, 7.0	37.2, 41.9
Q1, Q3	0.86, 5.57	2.50, 5.50	3.0, 7.0	50.0, 80.0	0.55, 0.81	3.0, 7.0	34.5, 44.2

1 year, treatment course remained systemic treatment only (*n* = 38)							
	BPI Pain Interference	BPI Pain Severity	Worst Pain	EQ-5D VAS	EQ-5Dindex	Worst Stiffness	PROMIS
Completed PROs	*n* = 28	*n* = 30	*n* = 30	*n* = 30	*n* = 29	*n* = 29	*n* = 30
Median	2.79	3.63	4.5	67.5	0.72	4.0	41.6
95% CI	1.29, 5.14	2.00, 5.25	2.0, 6.0	55.0, 80.0	0.65, 0.82	3.0, 6.0	37.5, 42.7
Q1, Q3	0.79, 5.50	1.25, 5.25	2.0, 7.0	50.0, 80.0	0.63, 0.82	2.0, 6.0	36.2, 44.5

Treatment course changed from systemic treatment only (*n* = 6)							
	BPI Pain Interference	BPI Pain Severity	Worst Pain	EQ-5D VAS	EQ-5Dindex	Worst Stiffness	PROMIS
Completed PROs	*n* = 6	*n* = 6	*n* = 6	*n* = 6	*n* = 6	*n* = 6	*n* = 6
Median	5.93	6.38	7.5	52.5	0.54	7.5	33.2
95% CI	3.14, 9.14	2.75, 7.75	5.0, 9.0	20.0, 75.0	-0.31, 0.80	2.0, 9.0	20.8, 42.8
Q1, Q3	3.57, 6.57	5.25, 7.00	6.0, 9.0	25.0, 70.0	0.16, 0.72	6.0, 9.0	28.7, 39.3

Unknown treatment (*n* = 1)							
2 years, treatment course remained on systemic treatment only (*n* = 30)^a^							
	BPI Pain Interference	BPI Pain Severity	Worst Pain	EQ-5D VAS	EQ-5Dindex	Worst Stiffness	PROMIS
Completed PROs	*n* = 22	*n* = 22	*n* = 22	*n* = 21	*n* = 21	*n* = 21	*n* = 21
Median	3.15	3.00	6.5	65.0	0.65	6.0	38.4
95% CI	1.00, 4.43	2.25, 5.50	3.0, 7.0	60.0, 80.0	0.58, 0.82	2.0, 7.0	34.7, 43.6
Q1, Q3	0.57, 4.43	1.00, 5.50	2.0, 7.0	60.0, 80.0	0.58, 0.82	2.0, 7.0	34.7, 43.6

Treatment course changed from systemic treatment only (*n* = 6)							
Completed PROs	*n* = 6	*n* = 6	*n* = 6	*n* = 6	*n* = 6	*n* = 6	*n* = 6
Median	4.07	4.13	6.0	77.5	0.72	5.5	39.9
95% CI	0, 7.14	0, 6.75	0, 7.0	50.0, 95.0	0.57, 1	0, 7.0	32.1, 41.6
Q1, Q3	0.86, 7.14	2.25, 5.50	3.0, 7.0	60.0, 90.0	0.60, 0.86	2.0, 7.0	35.1, 41.5

Surgery only, baseline (*n* = 39)							
	BPI Pain Interference	BPI Pain Severity	Worst Pain	EQ-5D VAS	EQ-5Dindex	Worst Stiffness	PROMIS
Completed PROs	*n* = 38	*n* = 38	*n* = 38	*n* = 38	*n* = 38	*n* = 37	*n* = 38
Median	2.72	4.38	4.5	75.0	0.74	6.0	40.4
95% CI	1.86, 5.00	1.75, 6.00	4.0, 7.0	65.0, 80.0	0.58, 0.79	5.0, 7.0	38.3, 44.2
Q1, Q3	1.14, 5.71	1.25, 6.25	2.0, 7.0	60.0, 85.0	0.50, 0.81	2.0, 8.0	36.2, 45.1

1 year, treatment course remained surgery only (*n* = 32)							
	BPI Pain Interference	BPI Pain Severity	Worst Pain	EQ-5D VAS	EQ-5Dindex	Worst Stiffness	PROMIS
Completed PROs	*n* = 19	*n* = 20	*n* = 20	*n* = 21	*n* = 20	*n* = 22	*n* = 22
Median	3.29	3.75	6.0	80.0	0.72	7.0	44.1
95% CI	1.43, 5.14	3.00, 5.00	4.0, 7.0	60.0, 85.0	0.66, 0.81	4.0, 7.0	36.3, 48.4
Q1, Q3	1.00, 5.14	3.00, 5.50	4.0, 7.5	60.0, 85.0	0.64, 0.81	3.0, 7.0	35.7, 48.4

Changed from surgery only (*n* = 0)							
Unknown treatment (*n* = 7)							
2 years, treatment course remained surgery only (*n* = 30)^a^							
	BPI Pain Interference	BPI Pain Severity	Worst Pain	EQ-5D VAS	EQ-5Dindex	Worst Stiffness	PROMIS
Completed PROs	*n* = 21	*n* = 21	*n* = 21	*n* = 22	*n* = 21	*n* = 20	*n* = 22
Median	2.43	2.50	4.0	82.5	0.81	4.5	43.5
95% CI	0.29, 3.71	1.00, 4.00	2.0, 5.0	75.0, 85.0	0.71, 0.83	1.0, 6.0	39.8, 46.9
Q1, Q3	0.29, 3.71	1.00, 4.00	2.0, 5.0	75.0, 85.0	0.71, 0.83	1.0, 6.0	38.6, 46.9
Changed from surgery only (*n* = 0)							
Unknown treatment (*n* = 2)^a^							

Scale:

BPI Pain Severity: NRS from 0 to 10, 0 = no pain, 10 = pain as bad as you can imagine.

BPI Pain Interference: NRS from 0 to 10, 0 = does not interfere, 10 = completely interferes.

Worst Stiffness: NRS from 0 to 10, 0 = no stiffness, 10 = stiffness as bad as you can imagine.

EQ-5D VAS: NRS from 0 to 100, 0 = worst health you can imagine, 100 = best health you can imagine.

EQ-5D index: NRS from 0 to 10, 0 = worst imaginable health state, 10 = best imaginable health state.

PROMIS: NRS from 0 to 100, 0 = worst health you can imagine, 100 = best health you can imagine.

^a^Patient population at year 2 comes from a sub-group of patients who remained no treatment at year 1.

Abbreviations: BPI, Brief Pain Inventory; CI, confidence interval; EQ-5D VAS, EuroQol-5 Dimension visual analog scale; NRS, numerical rating scale; PRO, patient-reported outcome; PROMIS, Patient-Reported Outcomes Measurement Information System; Q, quartile; SD, standard deviation.

#### Systemic Treatment at Baseline

**Figure 3.**
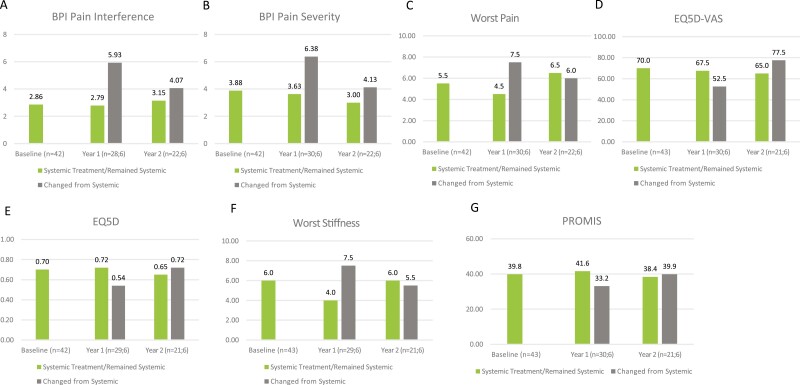
QoL related to systemic treatment at baseline assessed by the following PROs: (**A**) BPI Pain Interference; (**B**) BPI Pain Severity; (**C**) Worst Pain; (**D**) EQ-5D VAS; (**E**) EQ-5D; (**F**) Worst Stiffness; and (**G**) PROMIS scores at baseline, 1-year, and 2-year follow-ups. BPI = brief pain inventory; EQ-5D VAS = EuroQol-5 Dimension visual analog scale; PRO = patient-reported outcomes; PROMIS = Patient-Reported Outcomes Measurement Information System; QoL, quality of life. Calculated as median scores.

This section compares patients who were on systemic treatment at baseline and remained on systemic treatment throughout the 2-year observation period versus patients who changed from systemic treatment to a different treatment strategy in years 1 and/or 2. For patients treated with systemic treatment at baseline, numerically favorable scores were observed in patients who remained on systemic treatment compared with patients who switched to a different treatment strategy at year 1: median BPI Pain Interference (*n* = 28, 2.79 vs. *n* = 6, 5.93; Table 2; Fig. 3A), BPI Pain Severity (*n* = 30, 3.63 vs. *n* = 6, 6.38; Table 2; Fig. 3B), Worst Pain (*n* = 30, 4.5 vs. *n* = 6, 7.5; Table 2; Fig. 3C), Worst Stiffness (*n* = 29, 4.0 vs. *n* = 6, 7.5; Table 2; Fig. 3F) EQ-5D index score (*n* = 29, 0.72 vs. *n* = 6, 0.54; Table 2; Fig. 3E), EQ-5D VAS (*n* = 30, 67.5 vs. *n* = 6, 52.5; Table 2; Fig. 3D) and PROMIS (*n* = 30, 41.6 vs. *n* = 6, 33.2; Table 2; Fig. 3G). At year 2, numerically improved median BPI Pain Interference (*n* = 22, 3.15 vs. *n* = 6, 4.07; Table 2; Fig. 3A), and BPI Pain Severity (*n* = 22, 3.00; vs. *n* = 6, 4.13; Table 2; Fig. 3B) scores were seen in patients who remained on systemic treatment as compared with those who changed from systemic treatment. In contrast, EQ-5D VAS were numerically favorable at year 2 in patients who changed from systemic treatment (*n* = 6, 77.5) as compared with those who remained on systemic treatment (*n* = 21, 65.0; Table 2; Fig. 3D). No changes were observed in PROMIS (*n* = 21, 38.4 vs. *n* = 6, 39.9; Table 2; Fig. 3G), EQ-5D index score (*n* = 21, 0.65 vs. *n* = 6, 0.72; Table 2; Fig. 3E), and Worst Stiffness (*n* = 21, 6.0 vs. *n* = 6, 5.5; Table 2; Fig. 3F) scores.

#### Surgery at Baseline

Regarding patients who had surgery at baseline, no patients changed treatment course over the 2-year observation period. BPI Pain Interference was similar at baseline (*n* = 38, 2.72) and 2-year follow-up (*n* = 21, 2.43) (Table 2; Supplementary Fig. S3A). BPI Pain Severity scores decreased from baseline (*n* = 38, 4.38) to the 2-year visit (*n* = 21, 2.50). EQ-5D index (*n* = 38, 0.74 vs. *n* = 21, 0.81; Table 2; Supplementary Fig. S3B) and EQ-5D VAS (*n* = 38, 75.0 vs. *n* = 22, 82.5; Table 2; Supplementary Fig. S3D) scores showed a slight numerical increase between baseline and the 2-year visit. Worst Pain decreased between years 1 (*n* = 20, 6.0) and 2 (*n* = 21, 4.0; Table 2; Supplementary Fig. S3C). An increase in EQ-5D index score was observed from years 1 (*n* = 20, 0.72) to 2 (*n* = 21, 0.81; Table 2; Supplementary Fig. S3E). Worst Stiffness scores numerically decreased (*n* = 22, 7.0 vs. *n* = 20, 4.5; Table 2; Supplementary Fig. S3G) from the year-1 visit to the year-2 visit. PROMIS scores at baseline (*n* = 38, 40.4), years 1 (*n* = 22, 44.1) and 2 (*n* = 22, 43.5; Table 2; Supplementary Fig. S3F) were unchanged in the 2-year observation period.

#### PROs Based on Treatment Strategies on an Individual Patient Basis

From baseline through the 2-year follow-up visit, patients who remained as wait-and-see and surgery showed an improvement in BPI Pain Interference (Supplementary Fig. S4A). No changes over the 2-year observation period were seen in any of the other PROs, BPI Pain Severity (Supplementary Fig. S5A–S5D), Worst Stiffness (Supplementary Fig. S6A–S6D), EQ-5D index score (Supplementary Fig. S7A–S7D), EQ-5D VAS (Supplementary Fig. S8A–S8D), PROMIS (Supplementary Fig. S9A–S9D) for patients who either remained on a specific treatment strategy (wait-and-see, systemic, surgery) or for those who changed treatment strategy during the 2-year observation period.

## Discussion

TOPP represents the first and largest prospective, international, multicenter disease registry for D-TGCT, and includes 176 patients, demonstrating that conducting collaborative observational studies for a rare tumor is feasible. Due to the rarity of the disease, the current TGCT literature contains predominantly small, retrospective cohort studies, including heterogeneous data.^28^ In previous analyses, the TOPP registry described the impact of TGCT on PROs from a baseline snapshot,^25^ and subsequently a picture of the treatment journey of D-TGCT patients as a 2-year observational follow-up was reported.^26^ This analysis is the first to describe the impact of the disease on PROs as a 2-year follow-up based on treatment strategies.

Health-related QoL (HRQoL) is a key measure for the effectiveness and cost effectiveness of healthcare interventions and is used to evaluate the impact of the disease on the patients’ QoL assessment; however, HRQoL assessment in sarcoma is challenging due to the diversity of the disease and tumor location.^29^ Since D-TGCT is a locally aggressive disease affecting large joints and tendon sheaths, it is associated with functional impairment and negatively impacts patients’ QoL.^3,30^ Specifically, pain, swelling, stiffness, and reduced range of motion are considered hallmarks of the disease as they are the most common and critical symptoms experienced by patients with TGCT. As disease extent and the type of treatment approach might differently affect physical function and QoL, PRO assessments of disease symptoms, physical function, and HRQoL can serve as a valuable tool to support the relevance of primary endpoints in clinical trials.^8,31^

PRO measurements and QoL evaluation are therefore included as secondary outcome measures in most clinical trials, since it has been demonstrated that improvement of physical function and QoL were important treatment outcomes for patients with D-TGCT.^31-34^ Specifically, PROMIS-PF and Worst Stiffness scores were included in ENLIVEN to validate reduction in tumor size was meaningful to patients.^33^ As only changes of ≥3 score units for physical function are deemed meaningful by patients with D-TGCT, only minimal improvement was observed from baseline through the 2-year visit.

In this prospective analysis, there was a high patient compliance with PRO questionnaire completion. At baseline, the completion rate ranged from 93.8% (Worst Stiffness) to 96.0% (PROMIS, EQ-5D), and at the 2-year follow-up visit, most patients (>80%) had completed the questionnaires, demonstrating a high acceptance and interest for the study of this rare disease.

As D-TGCT severely affects the patients’ QoL and is responsible for pain and various degrees of limitations sustained over time, this plays a major role in the choice of the treatment strategy. As optimal treatment strategies remain to be elucidated, the multimodal treatment of D-TGCT patients is complex and depends on factors, such as tumor status at the time of assessment, previous treatment history, the clinical background of the treating physician, and information on PROs. At the baseline visit, different treatment approaches, mainly wait-and-see, surgery, or systemic treatment, were undertaken, and the treatment was monitored during the first and the second year of the observation period. For patients who started at baseline with no treatment, remaining Off-Treatment over the 2 years resulted in numerically better PRO scores. Specifically, lower scores were seen for the BPI Pain Interference, BPI Pain Severity, Worst Stiffness, and Worst Pain scales. As certain patients did not develop pain, there was no need to change from Off-Treatment to an active treatment strategy. Only in the instance of pain and discomfort complaints was a change to an On-Treatment strategy needed. For patients who started at baseline with systemic treatment (mainly pexidartinib or imatinib), remaining with systemic treatment over the 2 years resulted in numerically lower BPI Pain Interference and BPI Pain Severity scores, and higher EQ-5D index and EQ-5D VAS scores at year 1 compared with patients who switched treatment strategy. All patients who underwent surgery at baseline had no need for a different treatment within the 2-year follow-up visit; this confirms the disease’s biological behavior, which has a low growth rate and rarely presents with progressive disease and significant complaints within the first 2 years.^15^ Numerically improved PRO scores for BPI Pain Interference and BPI Pain Severity as well as for Worst Stiffness at the 2-year visit compared to baseline were observed.

Of note, at baseline, numerically favorable PROs were observed in Off-Treatment patients compared with patients in the systemic treatment and surgery groups. This might reflect a different patient population in terms of size, rate of relapse after previous treatment, or time from diagnosis. In D-TGCT patients, there is always a risk of aggravating symptoms by active treatment (ie, surgery), thus remaining Off-Treatment may be beneficial and have a better risk-to-benefit reward regarding PROs. The PROs recapitulate potential symptom triggers for treatment decisions. They also highlight the potential deterioration of the symptom load depending on specific therapeutic interventions, such as repetitive surgery. More specifically, a further deterioration of QoL was seen at 1 year, which was in large part due to D-TGCT treatment (primarily surgical intervention). Following systemic treatment, an association between tumor shrinkage and improvement in PROMIS-PF, pain relief, and Worst Stiffness scores have been reported.^35,36^ This emphasizes the need for a multidisciplinary approach in which systemic therapy may be a valuable treatment option prior to and/or after surgical resection.^37^

Most of the actual D-TGCT treatment was performed during the first year of the observation period, and the decision for a particular treatment was made taking several factors into account, which included the treating physicians’ clinical experience and PRO. PRO analyses demonstrated that D-TGCT severely affects the patients’ QoL and is responsible for pain and various degrees of limitations sustained over time, affecting the choice of the treatment strategy. Further deterioration of QoL was seen after 1 year of follow-up after surgery, which was mainly observed (and possibly caused) by D-TGCT treatment itself (primarily surgical intervention). Thus, the PRO measurements illustrate the continued heavy *disease* effect associated with D-TGCT, but also the potential *treatment* effect with lower PRO scores after surgery.

These findings indicate how PROs are relevant to show clinical improvement in patients with D-TGCT and perhaps warrant further assessment in larger patient populations to make treatment decisions in the future. As current literature lacks treatment guidelines and does not present relevant clinical findings that support clinical decision making, creating insight into such important factors can be of great value in optimizing treatment strategies focusing on the most significant individual outcome measure.

### Limitations

As this project is an observational study, the quality of the collected data has not been 100% verified. Due to the COVID-19 pandemic, the planned on-site monitoring visits in the United States could not be performed. Instead of on-site monitoring visits, more frequent and detailed remote monitoring visits were performed for these sites. Follow-up data for all enrolled patients were regularly monitored remotely, with additional review to minimize potential errors in data acquisition and reporting. The study spanned only 2 years, although it provides an entire overview of the patient journey in a slowly growing disease that can have effects for decades. Both patients in clinical studies and expanded access programs were allowed to enter the study. The study sites are tertiary sarcoma centers that include a difficult-to-treat population, and this study does not allow for analysis of the entire spectrum of TGCT. One-third of the patients (*n* = 61/176, 34.7%) were enrolled from 2 orthopedic sites in the Netherlands. The results of this study should be evaluated with care, as they might be prone to potential data bias. There remains a lack of systemic treatment options with only 1 agent approved in the United States and none in Europe. As the majority of the patients remained on the same treatment strategy, there were small numbers in some of the groups pertaining to patients that switched treatment courses. In turn, there is no statistical comparison, with the findings being descriptive.

## Conclusions

The present study underscores how D-TGCT has a major impact on various aspects of patients’ QoL and is responsible for pain and various degrees of limitations sustained over time, impacting the selection of treatment strategy. Patients who remained on systemic treatment showed a numerical improvement in PRO measurements compared with those who stopped their treatment. QoL was reduced at baseline and over time compared to QoL of a healthy population in all groups (ie, type of treatment strategy), illustrating the need for novel approaches in this population. The findings from the current analysis emphasize the relevance of PROs in TGCT trials. As novel treatments for D-TGCT are being actively studied, this analysis represents a benchmark and should be considered in future studies, including CSF1R inhibitors and surgical trials.

## Supplementary Material

oyad011_suppl_Supplementary_Figure_S1

oyad011_suppl_Supplementary_Figure_S2

oyad011_suppl_Supplementary_Figure_S3

oyad011_suppl_Supplementary_Figure_S4

oyad011_suppl_Supplementary_Figure_S5

oyad011_suppl_Supplementary_Figure_S6

oyad011_suppl_Supplementary_Figure_S7

oyad011_suppl_Supplementary_Figure_S8

oyad011_suppl_Supplementary_Figure_S9

oyad011_suppl_Supplementary_Table_S1

## Data Availability

De-identified individual participant data and applicable supporting clinical trial documents may be available upon request at https://vivli.org/ourmember/daiichi-sankyo//. In cases where clinical trial data and supporting documents are provided pursuant to our company policies and procedures, Daiichi Sankyo, Inc., will continue to protect the privacy of our clinical trial participants. Details on data sharing criteria and the procedure for requesting access can be found at this web address: https://www.clinicalstudydatarequest.com/Study-Sponsors/Study-Sponsors-DS.aspx.
