# Supplementary material for: Tenosynovial Giant Cell Tumor Observational Platform Project (TOPP) Registry: A 2-Year Analysis of Patient-Reported Outcomes and Treatment Strategies
Source: Oncologist. 2023 Mar 3;28(6):e425–35. doi: 10.1093/oncolo/oyad011 (PMC10243766; doi:10.1093/oncolo/oyad011)
Supplement: oyad011_suppl_Supplementary_Figure_S1 [file oyad011_suppl_supplementary_figure_s1.docx]

**Supplemental online Figure 1.** Patient eligibility.

Abbreviations: APS = All-documented Patient Set; BAS = Baseline Analysis Set;
FAS = Full Analysis Set; ICF = Informed Consent Form.
