# Supplementary figures and images for: Tenosynovial Giant Cell Tumor Observational Platform Project (TOPP) Registry: A 2-Year Analysis of Patient-Reported Outcomes and Treatment Strategies
Source: Oncologist. 2023 Mar 3;28(6):e425–35. doi: 10.1093/oncolo/oyad011 (PMC10243766; doi:10.1093/oncolo/oyad011)

**Supplemental online Figure 2.** Breakdown of t treatment and treatment plans at Baseline.


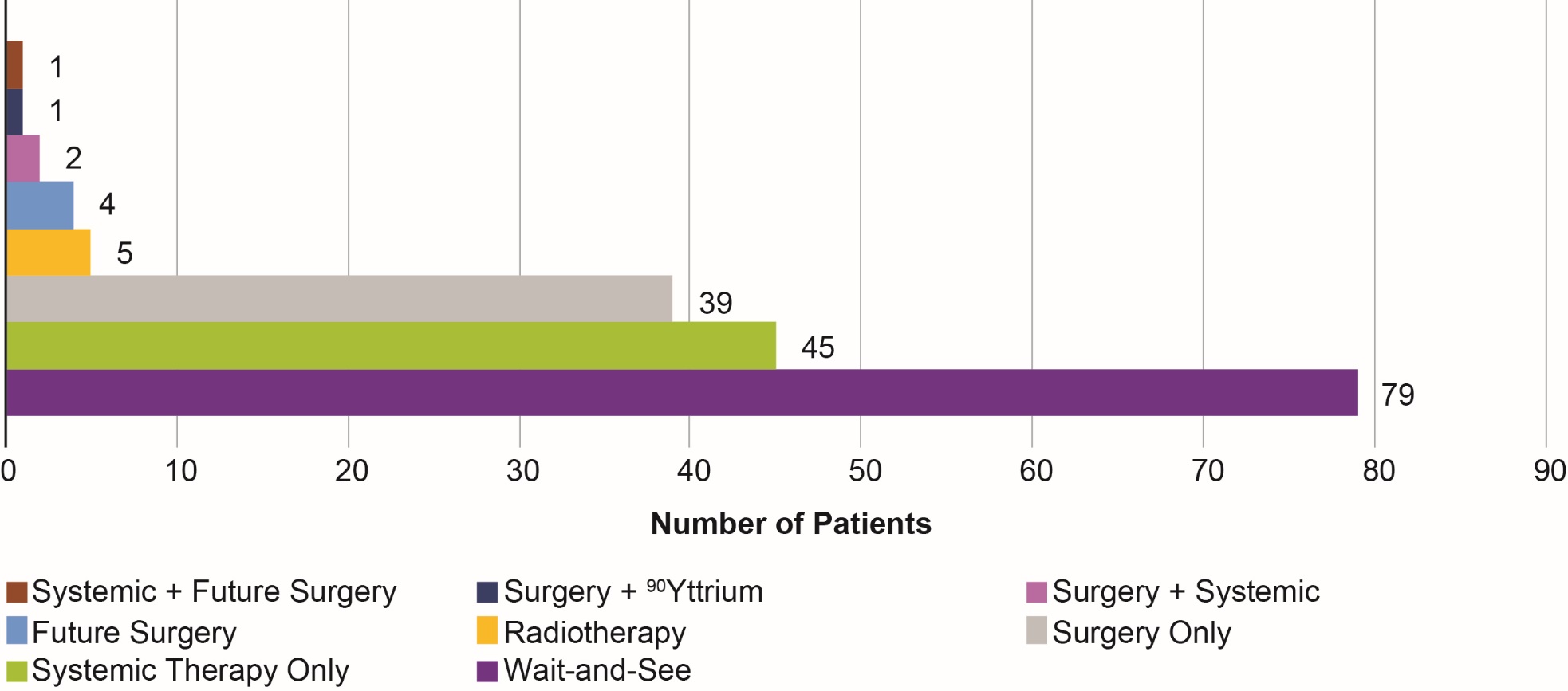

Supplement: oyad011_suppl_Supplementary_Figure_S2 [file oyad011_suppl_supplementary_figure_s2.docx]
