# Supplementary material for: Tenosynovial Giant Cell Tumor Observational Platform Project (TOPP) Registry: A 2-Year Analysis of Patient-Reported Outcomes and Treatment Strategies
Source: Oncologist. 2023 Mar 3;28(6):e425–35. doi: 10.1093/oncolo/oyad011 (PMC10243766; doi:10.1093/oncolo/oyad011)
Supplement: oyad011_suppl_Supplementary_Figure_S3 [file oyad011_suppl_supplementary_figure_s3.docx]

**Supplemental online Figure 3.** Quality of life for patients undergoing surgery at Baseline, and changes at Year 1 and Year 2 of observation assessed by the following PROs: (A) BPI Pain Interference; (B) BPI Pain Severity; (C) Worst Pain; (D) EQ-5D VAS;
(E) EQ-5D; (F) Worst Stiffness; and (G) PROMIS scores. Calculated as median scores.

Abbreviations: BPI = brief pain inventory; EQ-5D VAS = EuroQol-5 Dimension visual analog scale; PRO = patient reported outcomes; PROMIS = Patient-Reported Outcomes Measurement Information System.
