# Supplementary material for: Tenosynovial Giant Cell Tumor Observational Platform Project (TOPP) Registry: A 2-Year Analysis of Patient-Reported Outcomes and Treatment Strategies
Source: Oncologist. 2023 Mar 3;28(6):e425–35. doi: 10.1093/oncolo/oyad011 (PMC10243766; doi:10.1093/oncolo/oyad011)
Supplement: oyad011_suppl_Supplementary_Figure_S6 [file oyad011_suppl_supplementary_figure_s6.docx]

**Supplemental online Figure 6.** Worst Stiffness scores at Baseline, 1-Year, and 2-Years in patients based on treatment strategy:
(A) Remained Wait-and-See; (B) Remained Surgery; (C) Remained Systemic; (D) Changed Treatment Strategy.

**Remained Wait-and-See** **Remained Surgery**  ****

**Remained Systemic Treatment** **Changed Treatment Strategy**
