# Supplementary material for: Tenosynovial Giant Cell Tumor Observational Platform Project (TOPP) Registry: A 2-Year Analysis of Patient-Reported Outcomes and Treatment Strategies
Source: Oncologist. 2023 Mar 3;28(6):e425–35. doi: 10.1093/oncolo/oyad011 (PMC10243766; doi:10.1093/oncolo/oyad011)
Supplement: oyad011_suppl_Supplementary_Table_S1 [file oyad011_suppl_supplementary_table_s1.docx]

**Supplemental online Table 1.** Demographics and treatments at Baseline of TOPP.

| **Features** | ***N* = 176** |
| --- | --- |
| **Age at enrollment, mean, years ± SD Age at diagnosis, mean, years ± SD** | 44 ±14.3 38 ±14.6 |
| **Gender, *n* (%)** Female Male | 108 (61) 68 (39) |
| **Tumor Site, *n* (%)** Knee Ankle Hip Shoulder Foot Elbow Wrist Hand Temporomandibular | 120 (68) 18 (10) 12 (7) 8 (5) 7 (4) 4 (2) 3 (2) 3 (2) 1 (1) |
| **Treatment Plan at Baseline, *n* (%)** Off-Treatment  On-Treatment | 79 (45) 97 (55) |

Abbreviations: SD, standard deviation; TGCT, tenosynovial giant cell tumor; TOPP, TGCT Observational Platform Project.
